# CRP and D-dimer for risk stratification of cerebral infarction in children with *Mycoplasma pneumoniae* pneumonia: a retrospective study

**DOI:** 10.3389/fneur.2026.1771225

**Published:** 2026-04-20

**Authors:** Huan Deng, Tian Lv, Feng Liu, Deyu Zhao, Xia Huang, Dezhi Qiu

**Affiliations:** 1 Department of Respiratory Medicine, Children’s Hospital of Nanjing Medical University, Nanjing, China; 2 Department of Pediatrics, Punan Branch of Renji Hospital, Shanghai Jiao Tong University School of Medicine (Punan Hospital in Pudong New District), Shanghai, China; 3 Department of Neurosurgery, Children’s Hospital of Nanjing Medical University, Nanjing, China

**Keywords:** cerebral infarction, C-reactive protein, D-dimer, *Mycoplasma pneumoniae*, risk stratification

## Abstract

**Background:**

Cerebral infarction (CI) is a rare but severe complication of *Mycoplasma pneumoniae* pneumonia (MPP) in children.

**Objective:**

This study aimed to explore the clinical characteristics and factors associated with CI in pediatric patients with MPP.

**Methods:**

A retrospective cohort study of children with MPP was conducted between 2019 and 2023. Clinical characteristics, laboratory findings, and imaging results were collected and analyzed. Propensity score matching (PSM) was applied to reduce potential confounding, and Firth’s penalized logistic regression was performed as an exploratory analysis to assess associations between selected variables and CI.

**Results:**

Among 2,947 children diagnosed with MPP, 9 (0.3%) developed CI. In the matched cohort, higher levels of C-reactive protein (CRP) and D-dimer were significantly associated with the occurrence of CI [CRP: odds ratio (OR) = 1.08, *p* = 0.003; D-dimer: OR = 1.00026, *p* = 0.007]. Receiver operating characteristic (ROC) analysis demonstrated that the combination of CRP and D-dimer showed good discriminatory performance for CI, with an area under the curve of 0.920 (*p* < 0.001).

**Conclusion:**

Elevated CRP and D-dimer levels were associated with CI in children with MPP and may be useful for risk stratification. These findings highlight the potential interplay between inflammation and coagulation in CI complicating MPP. Further multicenter studies with larger sample sizes are required to validate these results and clarify their clinical utility.

## 1 Background

*Mycoplasma pneumoniae* (MP) is a leading cause of community-acquired pneumonia in children, with regional outbreaks occurring globally every 3 to 7 years. Following the relaxation of COVID-19 restrictions, a resurgence of pediatric respiratory infections has been observed, with several European countries, including Denmark, reporting an increase in MP-related respiratory diseases (1). This trend has also been confirmed by a global surveillance study involving 24 countries across four continents (2). In China, pediatric respiratory illnesses—particularly *Mycoplasma pneumoniae* pneumonia (MPP)—have risen significantly since 2023, with a growing proportion of severe cases (3).

As many as 25% of individuals with MPP may experience extrapulmonary complications, which can arise at varying times after the onset of respiratory symptoms, or even in the absence of respiratory illness (4). Thrombosis is one such extrapulmonary manifestation, although it is relatively rare in children with MPP. In a study conducted at Tianjin Children’s Hospital from January 2016 to April 2020, the incidence of thrombosis in MPP was 0.17% (5). Thrombosis associated with MPP can occur in various parts of the body. Neurologic complications related to MP infection are uncommon, occurring in approximately 6 to 7% of cases (6), with cerebral infarction (CI) being even rarer. Without timely diagnosis and treatment, these complications can lead to severe sequelae and even life-threatening outcomes. A follow-up at Tianjin Children’s Hospital on three children with MPP-induced cerebral embolism revealed that, despite aggressive thrombolysis and anticoagulation, all survivors experienced long-term neurological sequelae (5). However, there are few reports both domestically and internationally on cerebral infarction caused by MP infection, and data on the clinical characteristics of such cases remain limited.

In this retrospective study, we reviewed the clinical data of nine children with MPP complicated by CI at the Children’s Hospital of Nanjing Medical University between 2019 and 2023. Using propensity score matching (PSM), contemporaneous children with MPP without CI were selected as controls. By comparing clinical and laboratory characteristics between the two groups, this study aimed to explore factors associated with CI in children with MPP and to improve clinical awareness for early recognition of this rare but severe complication.

## 2 Methods

### 2.1 Study population

Clinical data of children diagnosed with MPP and admitted to the Children’s Hospital of Nanjing Medical University between January 2019 and December 2023 were retrospectively collected.

The inclusion criteria were as follows: (1) age between 28 days and 18 years; (2) presence of respiratory symptoms; (3) laboratory evidence of *Mycoplasma pneumoniae* infection, defined by positive MP antibodies and/or nucleic acid testing; and (4) radiographic evidence of pneumonia. The exclusion criteria were as follows: (1) co-infection with other pathogens; (2) history of head trauma; (3) pre-existing neurological disorders, including encephalitis, epilepsy, stroke, or other cerebrovascular diseases; and (4) incomplete medical records.

Clinical data, including age, sex, and pre-admission fever duration, were extracted from the electronic medical records. Laboratory parameters obtained within 24 h of admission included white blood cell (WBC) count, neutrophil-to-lymphocyte ratio (NLR), C-reactive protein (CRP), alanine aminotransferase (ALT), lactate dehydrogenase (LDH), and coagulation-related indices. Imaging data included chest imaging, brain computed tomography (CT), brain magnetic resonance imaging (MRI), and magnetic resonance angiography (MRA), when available.

CI was diagnosed based on the presence of acute neurological symptoms (including headache, focal motor deficits, facial paralysis, impaired consciousness, or epileptic seizures) together with radiological evidence of cerebral infarction on brain MRI and/or vascular abnormalities on MRA. Control patients were defined as children with MPP who did not develop CI within 60 days after disease onset.

### 2.2 Statistical analysis

Statistical analyses were performed using R version 4.5.1 and IBM SPSS Statistics version 26. Continuous variables are presented as mean ± standard deviation or median with interquartile range and were compared using the independent-samples *t*-test or Mann–Whitney *U* test, as appropriate. Categorical variables are expressed as counts (percentages) and compared using Fisher’s exact test. To reduce potential confounding, PSM was performed using the MatchIt package in R, with age, sex, and pre-admission fever duration included in the propensity score mode. Nearest-neighbor matching without replacement was conducted at a 1:8 ratio based on the logit of the propensity score, with a caliper width of 0.05 standard deviations. Sensitivity analyses were conducted using alternative matching strategies, including a smaller caliper width (0.02) and optimal matching, to evaluate the robustness of the results. After matching, laboratory variables were compared between groups. Given the limited number of outcome events, Firth’s penalized logistic regression was applied as an exploratory analysis to assess associations between selected laboratory indicators and CI. To avoid model overfitting, only a limited number of key variables were included in the regression models. Receiver operating characteristic (ROC) curve analysis was performed to evaluate the discriminatory performance of individual biomarkers and their combination. In addition, *E*-value analysis was performed to assess the robustness of the observed associations to potential unmeasured confounding. All statistical tests were two-sided, and a *p*-value <0.05 was considered statistically significant.

## 3 Results

**Figure 1:**
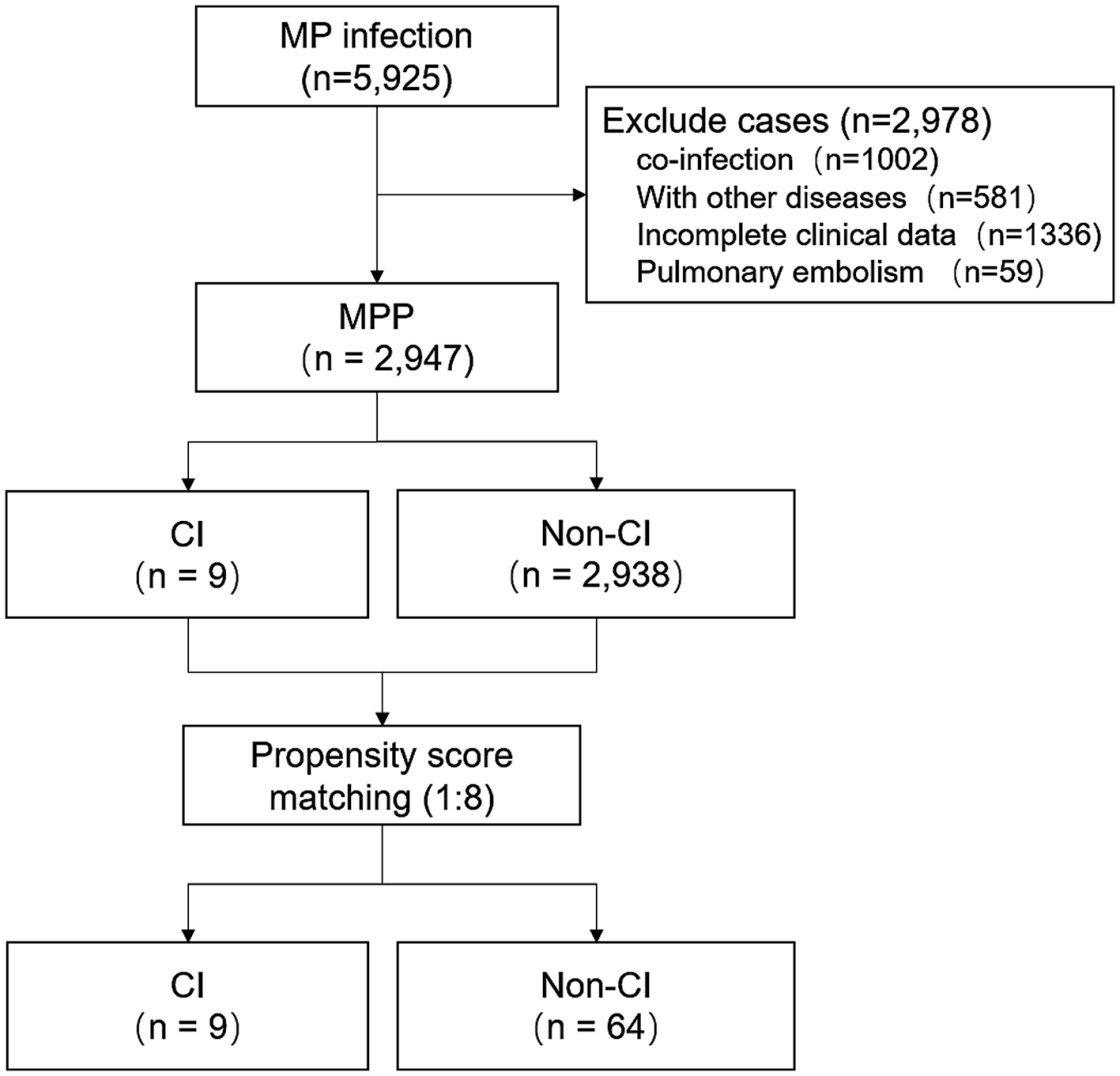
Flowchart of the study design. MPP, *Mycoplasma pneumoniae* pneumonia; CI, cerebral infarction.

A total of 5,925 patients with MPP were initially screened. After applying the inclusion and exclusion criteria, 2,947 patients were included in the final analysis, comprising 9 patients with CI and 2,938 patients without CI. PSM was then performed at a 1:8 ratio between the CI and non-CI groups, resulting in a matched cohort of 9 patients with CI and 64 patients without CI (Figure 1). Age, sex, and pre-admission fever duration were used as matching variables. After matching, the baseline characteristics of the two groups were substantially balanced, with acceptable balance across the matching covariates, and adequate overlap of propensity scores (Supplementary Figure 1).

### 3.1 Clinical manifestations and outcomes in children with MPP and CI

Among patients in the CI group, the mean age at onset was 7.5 years; there were 7 males and 2 females (Table 1; Supplementary Table 1). All patients presented with fever at disease onset. Neurological manifestations included abnormal muscle strength or tone in 5 patients, disturbance of consciousness in 2 patients, and epileptic seizures in 4 patients. The median interval from disease onset to the occurrence of CI was 9 days.

**Table 1:** *Mycoplasma pneumoniae* pneumonia (MPP) cohort.

Characteristics	Primary MPP cohort		*p*-value	Matched MPP cohort		*p*-value
	Non-CI (*n* = 2,938)	CI (*n* = 9)		Non-CI (*n* = 64)	CI (*n* = 9)	
Sex (*n*, %)						
Male	1,546 (52.6)	7 (77.8)	0.185	49 (76.6)	7 (77.8)	0.652
Female	1,392 (47.4)	2 (22.2)		15 (23.4)	2 (22.2)	
Age (years)	6.3 ± 2.8	7.5 ± 3.4	0.323	7.3 ± 2.8	7.5 ± 3.4	0.923
Pre-admission fever duration (days)	6.9 ± 3.7	4.9 ± 2.3	0.031	5.5 ± 4.2	4.9 ± 2.3	0.500
WBC (×10^9^/L)	8.8 (6.8–11.5)	11.8 (9.5–16.8)	0.035	10.0 (7.3–12.7)	11.8 (9.5–16.8)	0.144
NLR	2.9 (2.0–4.3)	11.1 (4.0–18.09)	<0.001	3.9 (2.1–6.0)	11.1 (4.0–18.09)	0.005
Platelets (×10^9^/L)	300.0 (230.0–388.0)	254.0 (157.5–343.5)	0.194	307.5 (235.3–394.8)	254.0 (157.5–343.5)	0.174
CRP (mg/L)	5.7 (2.9–14.6)	25.5 (13.0–60.3)	0.001	5.67 (1.4–12.3)	25.5 (13.0–60.3)	0.001
ALT (U/L)	14.0 (11.0–21.0)	19.0 (10.5–78.0)	0.235	14.5(11.0–26.5)	19.0 (10.5–78.0)	0.118
LDH (U/L)	326.0 (279.0–397.0)	606.0 (248.0–896.0)	0.151	311.5 (269.0–374.5)	606.0 (248.0–896.0)	0.129
Prothrombin time (s)	12.2 (11.5–13.0)	12.9 (11.3–13.5)	0.424	12.1 (11.4–13.0)	12.9 (11.3–13.5)	0.406
APTT (s)	31.4 (28.6–34.5)	28.0 (26.0–30.9)	0.015	31.7 (28.9–37.1)	28.0 (26.0–30.9)	0.023
Fibrinogen (g/L)	3.5 (3.0–3.9)	2.6(2.4–3.4)	0.013	3.3 (2.8–3.9)	2.6(2.4–3.4)	0.041
D-dimer (ng/mL)	227.0 (150.0–415.3)	4255.0 (756.0–9584.5)	<0.001	174.5 (125.5–284.8)	4255.0 (756.0–9584.5)	0.001

Statistics: *p*-values were calculated using the unpaired two-tailed Student’s *t*-test, the Mann–Whitney *U* test, or Fisher’s exact test. Continuous variables are presented as mean ± standard deviation or median (interquartile range), and categorical variables are presented as number (percentage). PSM was performed using nearest-neighbor matching with a caliper of 0.05.

APTT, activated partial thromboplastin time; ALT, alanine aminotransferase; CI, cerebral infarction; CRP, C-reactive protein; LDH, lactate dehydrogenase; MPP, *Mycoplasma pneumoniae* pneumonia; NLR, neutrophil-to-lymphocyte ratio; WBC, white blood cell count.

**Figure 2:**
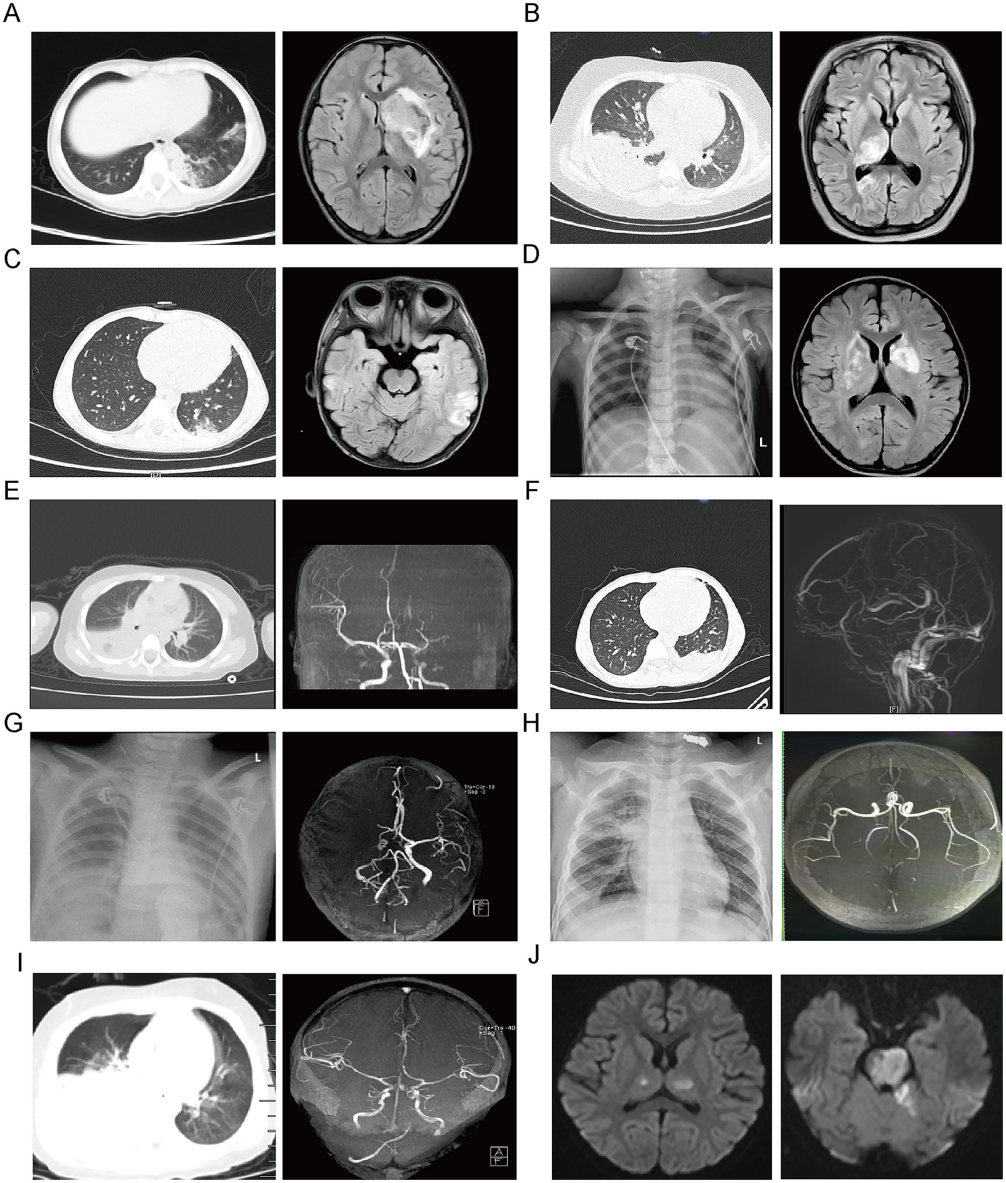
Radiologic manifestations in nine children with cerebral infarction (CI) complicating *Mycoplasma pneumoniae* pneumonia. **(A)** Patient 1: Chest CT showing left-sided pneumonia (left); brain MRI demonstrating abnormal signal intensity in the left frontoparietal cortex with involvement of the lateral ventricle (right). **(B)** Patient 2: Chest CT revealing large consolidation in the right lower lobe (left); brain MRI showing multiple irregular patchy lesions involving the right thalamus, right occipital lobe, and bilateral cerebellar hemispheres (right). **(C)** Patient 3: Chest CT showing right-sided pneumonia (left); brain MRI demonstrating abnormal signal intensity in the left temporo-occipital lobe (right). **(D)** Patient 4: Chest CT demonstrating bilateral pneumonia (left); brain MRI revealing multifocal abnormal signal intensities in the bilateral basal ganglia and cerebral peduncles (right). **(E)** Patient 5: Chest CT showing left-sided pneumonia with pleural effusion (left); magnetic resonance angiography (MRA) demonstrating non-visualization of the left anterior cerebral artery and middle cerebral artery and their branches (right). **(F)** Patient 6: Chest CT demonstrating consolidation in the left lower lobe (left); magnetic resonance venography showing non-visualization of the superior sagittal sinus and adjacent superficial cortical veins (right). **(G)** Patient 7: Chest radiograph showing right-sided pneumonia with pleural effusion (left); MRA demonstrating occlusion of the right middle cerebral artery (right). **(H)** Patient 8: Chest radiograph showing right upper- and lower-lobe pneumonia (left); MRA demonstrating absence of flow-related signal in the basilar artery and the P1 segments of the bilateral posterior cerebral arteries (right). **(I)** Patient 9: Chest CT showing right upper- and lower-lobe pneumonia (left); MRA demonstrating poor visualization of the distal basilar artery and non-visualization of the P1 segments of the bilateral posterior cerebral arteries (right). **(J)** Patient 9: Diffusion-weighted imaging demonstrating abnormal signal intensities in the thalamus (left), brainstem, and cerebellum (right).

The positivity rates of serum MP antibodies and MP DNA were both 100%, whereas MP nucleic acid was not detected in the cerebrospinal fluid of any patient. Chest imaging predominantly showed unilateral pneumonia or pneumonia mainly affecting one lung. Cranial imaging revealed lesions in the left hemisphere in 4 patients (44.4%), the right hemisphere in 2 patients (22.2%), and both hemispheres in 3 patients (33.3%). Lesions were distributed across multiple brain regions, including the cerebral hemispheres (1 patient), basal ganglia (4 patients), temporal lobe (4 patients), occipital lobe (2 patients), brainstem (2 patients), and cerebellum (2 patients). The affected vascular territories included the anterior cerebral artery (2 patients), middle cerebral artery (2 patients), internal carotid artery (1 patient), basilar artery with bilateral posterior cerebral arteries (2 patients), and superficial cerebral veins (1 patient) (Supplementary Table 1 and Figure 2).

Complete follow-up data were available up to 12 December 2025. Among the nine children with CI, three died, and the six survivors were left with neurological sequelae, including motor impairment, muscle weakness, or facial paralysis.

### 3.2 Inflammatory and coagulation characteristics associated with CI

The demographic and baseline clinical characteristics of the 2,938 children with MPP are summarized in Table 1. Compared with patients without CI, those with CI had significantly higher WBC counts, NLR, CRP, fibrinogen levels, activated partial thromboplastin time (APTT), and D-dimer levels (all *p* < 0.05), as well as a shorter pre-admission fever duration (*p* < 0.05) (Table 1). After PSM, the between-group differences in NLR, CRP, D-dimer, fibrinogen, and APTT remained statistically significant (Table 1).

### 3.3 Factors associated with CI in MPP

**Figure 3:**
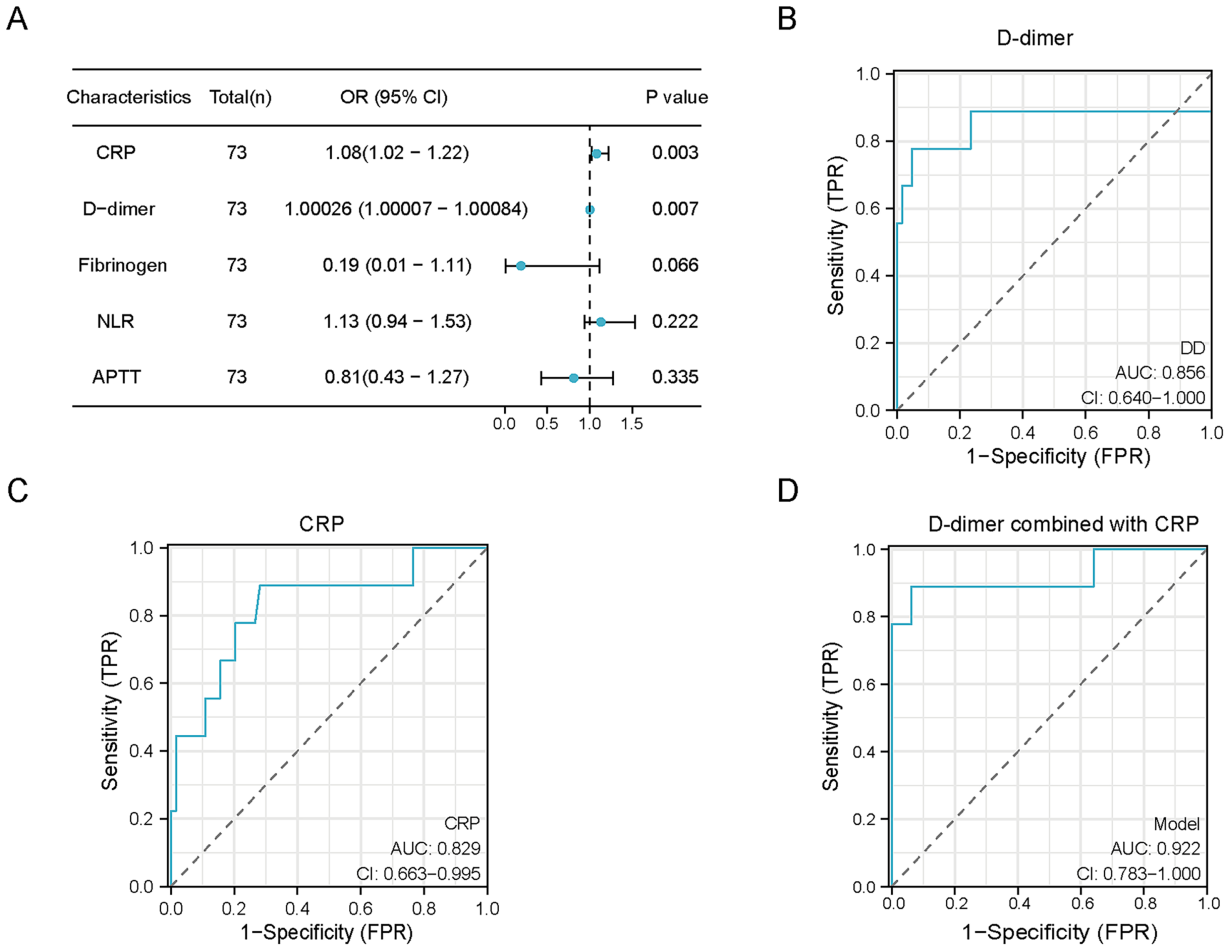
Associations and discriminatory performance of inflammatory and coagulation biomarkers for cerebral infarction (CI) complicating *Mycoplasma pneumoniae* pneumonia (MPP). **(A)** Forest plot showing odds ratios (ORs) and 95% confidence intervals (CIs) for the associations between selected laboratory biomarkers and CI in the propensity score–matched (PSM) cohort, assessed using Firth’s penalized logistic regression analysis. **(B)** Receiver operating characteristic (ROC) curve of D-dimer for discriminating CI. **(C)** ROC curve of C-reactive protein (CRP) for discriminating CI. **(D)** ROC curve of the combined model including CRP and D-dimer, showing improved discriminatory performance compared with either biomarker alone.

In the Firth penalized logistic regression analysis restricted to the matched cohort, CRP and D-dimer were significantly associated with CI among the evaluated biomarkers (NLR, CRP, D-dimer, fibrinogen, and APTT) (CRP: OR = 1.08, *p* = 0.003; D-dimer: OR = 1.00026, *p* = 0.007; Figure 3A). Sensitivity analyses using alternative matching strategies, including a stricter caliper and optimal matching, yielded consistent results (Supplementary Tables 2–5). *E*-value analysis showed that the observed association for CRP had an *E*-value of 1.37 (lower bound, 1.16), whereas the corresponding *E*-value for D-dimer was 1.02 (lower bound, 1.01). In receiver operating characteristic (ROC) analysis, both biomarkers individually showed good discriminatory performance for CI complicating MPP (CRP: AUC = 0.829, *p* < 0.001; D-dimer: AUC = 0.856, *p* = 0.001; Figures 3B,C). The combination of CRP and D-dimer further improved discriminatory performance, with an AUC of 0.920 (*p* < 0.001; Figure 3D).

## 4 Discussion

CI is a rare but potentially severe extrapulmonary complication of MPP. Consistent with previous reports (7, 8), CI in the present cohort occurred predominantly in school-aged boys, with a median interval of 9 days from disease onset to infarction. The neurological manifestations of MPP-related CI were heterogeneous, with both symptom patterns and severity varying according to patient age, severity of infection, and the vascular territory involved. Motor deficits were the most common clinical presentation, while central facial palsy, impaired consciousness, and epileptic seizures were also observed. Cranial magnetic resonance imaging was the primary diagnostic modality for identifying the location and extent of cerebral infarction.

The pathogenesis of MP–associated CI remains incompletely understood. Proposed mechanisms generally point to a transient hypercoagulable state that predisposes to vascular thrombosis, potentially triggered by direct endothelial injury, bacterial cytotoxins, or post-infectious immune responses (7, 8). Although sporadic reports have detected MP DNA in cerebrospinal fluid (9, 10), the uniformly negative PCR results observed in our cohort and in recent series (8), together with the finding that approximately half of CI events occurred after respiratory symptoms had resolved or were waning, suggest that most central nervous system complications are more likely immune-mediated rather than the result of direct microbial invasion. Under this framework, biological markers reflecting inflammation, immune-mediated vascular injury, and coagulation imbalance may be informative for identifying children at increased risk of CI. In line with this, our retrospective analysis showed that both CRP and D-dimer were significantly associated with cerebral infarction after propensity score matching and multivariable Firth’s penalized logistic regression analysis, supporting their potential value as risk stratification markers in children with MPP.

D-dimer is a fibrin degradation product, and its elevated level is closely related to thrombosis and activation of fibrinolysis. In MPP, increased D-dimer levels may reflect a hypercoagulable state and microvascular injury during the infectious process (11). In addition, MP infection may be associated with endothelial injury and immune-mediated vascular inflammation, which might further promote coagulation activation and microthrombus formation (6, 12). These changes may partly contribute to cerebral vascular occlusion and the development of CI. Previous studies have shown that changes in D-dimer levels are associated with disease severity and clinical outcomes in patients with MPP (11, 13, 14). Higher D-dimer levels may indicate more severe illness or an increased risk of thromboembolic complications in children (15), and D-dimer has been reported to be sensitive for identifying thromboembolic events in MPP (16, 17). Consistent with these findings, our results showed that elevated D-dimer levels were significantly associated with the occurrence of CI in children with MPP. However, D-dimer levels may also be elevated in a wide range of conditions, including infections, inflammatory disorders, malignancies, and refractory MPP (11, 18). Moreover, not all children with CI present with elevated D-dimer levels. Although a normal D-dimer value is often used to help exclude thrombotic disease in adults, thrombotic events have also been reported in children with normal D-dimer levels (18). In our cohort, one child had a D-dimer level of only 2 ng/mL, possibly because respiratory symptoms had already subsided at the time of admission. Although D-dimer was significantly associated with CI, the relatively small *E*-value suggests that further validation is warranted. Therefore, D-dimer should be interpreted in combination with other clinical and laboratory findings rather than used alone to establish or exclude a diagnosis of CI in children with MPP.

CRP is more than a nonspecific inflammatory marker in MPP and has been implicated in disease severity and coagulation abnormalities. Higher CRP levels have been shown to correlate with disease severity and prolonged radiological resolution in MPP (14, 19). Mechanistically, CRP has been reported to induce airway epithelial injury through the p38 MAPK–mitochondrial apoptosis pathway (20). In the vascular system, CRP has also been implicated in endothelial activation and prothrombotic responses, including upregulation of tissue factor expression and a shift toward a procoagulant phenotype, which may contribute to amplification of the coagulation cascade (21, 22). Consistent with these mechanistic observations, CRP levels were significantly associated with the occurrence of cerebral infarction (CI) in the present propensity score-matched cohort. Each 1 mg/L increase in CRP was associated with an approximately 8% higher odds of CI. Although serial CRP measurements may reflect both inflammatory burden and potential coagulation activation, CRP alone is insufficient for diagnosing CI. The clinical utility of CRP as part of a multimarker strategy for risk stratification warrants further investigation in larger, prospective studies.

This study has several limitations. CI is a rare complication of MPP, and the sample size in this study was limited. Consequently, the results of the Firth penalized logistic regression should be interpreted with caution and are regarded as hypothesis-generating rather than definitive. In addition, this was a single-center retrospective study, and other potential prognostic indicators were not included. Only commonly available clinical and laboratory variables were analyzed. Pre-existing neurological disorders may influence the risk of cerebral infarction through multiple mechanisms and could act as potential confounders. Previous studies have shown that early brain injury or neurological impairment may lead to increased vulnerability of the developing brain to subsequent insults, including hypoxic or ischemic injury (23–25). This heightened susceptibility may, to some extent, contribute to adverse cerebrovascular outcomes in children. In the present study, patients with known pre-existing neurological disorders were excluded to minimize potential confounding. However, residual confounding cannot be completely ruled out, and larger multicenter prospective studies are warranted to further validate and extend these findings.

## 5 Conclusion

In conclusion, elevated CRP and D-dimer levels were significantly associated with the occurrence of CI in children with MPP and showed good discriminatory performance in the matched cohort. These findings suggest that CRP and D-dimer may serve as potential risk stratification markers, although their clinical utility and generalizability require further validation in larger multicenter pediatric stroke registries.

## Data Availability

The original contributions presented in the study are included in the article/Supplementary material. Further inquiries can be directed to the corresponding author.
