# Supplementary material for: CRP and D-dimer for risk stratification of cerebral infarction in children with Mycoplasma pneumoniae pneumonia: a retrospective study
Source: Front Neurol. 2026 Apr 20;17:1771225. doi: 10.3389/fneur.2026.1771225 (PMC13137814; doi:10.3389/fneur.2026.1771225)
Supplement: Supplementary file 1 [file Supplementary_file_1.docx]

Supplementary Material

# Supplementary Figures and Tables

## Supplementary Figure


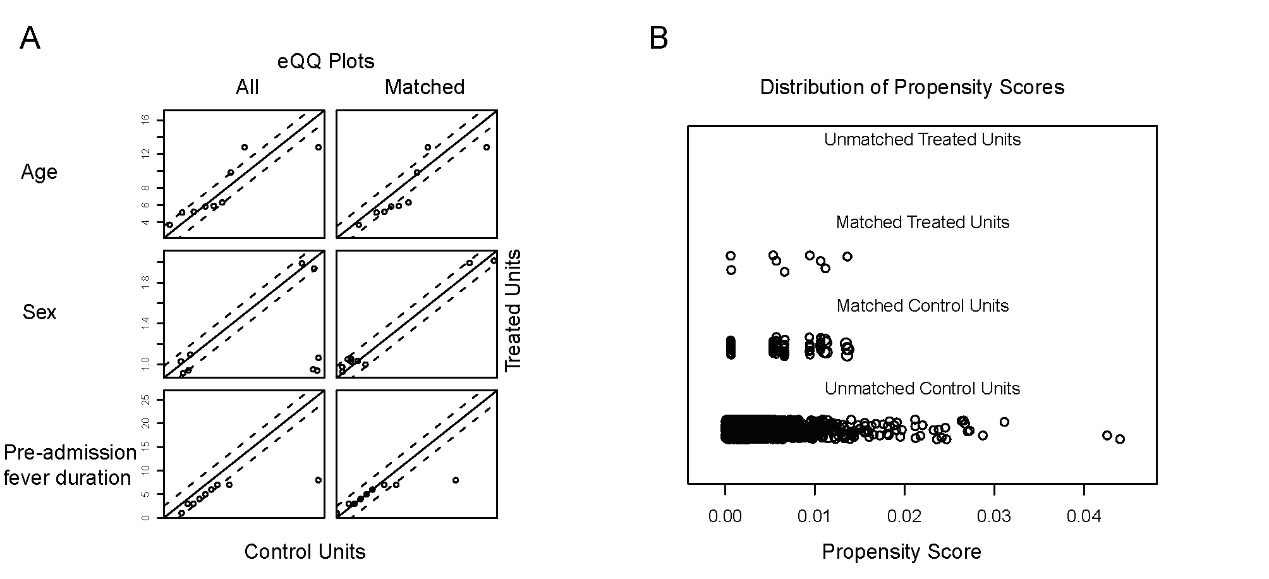


**Supplementary Figure 1.** Assessment of propensity score matching (PSM).
(A) Empirical quantile–quantile (eQQ) plots comparing the distributions of baseline covariates (age, sex, and pre-admission fever duration) between treated and control units before matching (All) and after matching (Matched). Closer alignment of points along the diagonal line after matching indicates improved covariate balance between groups.
(B) Distribution of propensity scores for treated and control units before and after matching. The increased overlap in propensity score distributions between matched treated and matched control units indicates improved comparability following propensity score matching.

Supplementary Tables

Table 1. Baseline characteristics of the matched cohort using propensity score matching with a caliper width of 0.02 for sensitivity analysis.

| Characteristics | Matched MPP cohort | | *P-value* |
| --- | --- | --- | --- |
|  | Non- CI  (n=58) | CI  (n=9) |  |
| Sex (n, %) |  |  |  |
| Male | 43(74.2) | 7 (77.8) | 0.590 |
| Female | 15(25.9) | 2(22.2) |  |
| Age (y) | 7.4 ± 2.8 | 7.5 ± 3.4 | 0.910 |
| Pre-admission fever duration (d) | 5.7 ± 4.3 | 4.9 ± 2.3 | 0.599 |
| WBC (×10^9^/L) | 10.0 (7.2–12.8) | 11.8 (9.5–16.8) | 0.144 |
| NLR | 3.8 (2.0–5.5) | 11.1 (4.0–18.09) | 0.005 |
| Platelets (×10^9^/L) | 307.5 (228.0–395.8) | 254.0 (157.5–343.5) | 0.174 |
| **CRP (mg/L)** | 5.7 (1.3–13.3) | 25.5 (13.0–60.3) | 0.001 |
| ALT (U/L) | 14.0(11.0–24.0) | 19.0 (10.5–78.0) | 0.118 |
| LDH (U/L) | 311.5 (269.0–377.0) | 606.0 (248.0–896.0) | 0.129 |
| PT (s) | 12.0 (11.4–12.9) | 12.9 (11.3–13.5) | 0.406 |
| **APTT (s)** | 31.7 (29.0–35.1) | 28.0 (26.0–30.9) | 0.023 |
| **Fibrinogen (g/L)** | 3.4 (2.9–3.9) | 2.6(2.4–3.4) | 0.041 |
| **D-dimer (ng/mL)** | 173.0 (124.8–274.3) | 4255.0 (756.0–9584.5) | 0.001 |

Statistics: P values were calculated using the unpaired two-tailed Student’s *t* test, the Mann–Whitney *U* test, or Fisher’s exact test. Continuous variables are presented as mean ± standard deviation or median (interquartile range), and categorical variables are presented as number (percentage). PSM was performed using nearest-neighbor matching with a caliper of 0.02. Abbreviations: APTT, activated partial thromboplastin time; ALT, alanine aminotransferase; CI, cerebral infarction; CRP, C-reactive protein; LDH, lactate dehydrogenase; MPP, Mycoplasma pneumoniae pneumonia; NLR, neutrophil-to-lymphocyte ratio; WBC, white blood cell count.

Table 2. Multivariable Firth’s penalized logistic regression analysis in the propensity score–matched cohort using a caliper width of 0.02.

| Characteristics | OR | 95% CI | P |
| --- | --- | --- | --- |
| **CRP** | 1.071 | 1.010 – 1.202 | **0.010** |
| NLR | 1.160 | 0.948 – 1.695 | 0.172 |
| APTT | 0.861 | 0.479 – 1.312 | 0.448 |
| **D-dimer** | 1.00024 | 1.00006 – 1.00065 | **0.010** |
| **Fibrinogen** | 0.207 | 0.015 – 1.051 | 0.058 |

Table 3. Multivariable Firth penalized logistic regression analysis of the propensity score–matched cohort using optimal matching

| Characteristics | Matched MPP cohort | | *P-value* |
| --- | --- | --- | --- |
|  | Non- CI  (n=72) | CI  (n=9) |  |
| Sex (n, %) |  |  |  |
| Male | 51(87.9) | 7(12.1) | 0.501 |
| Female | 21(91.3) | 2(8.7) |  |
| Age (y) | 8.3 ± 3.0 | 7.5 ± 3.4 | 0.481 |
| Pre-admission fever duration (d) | 5.3 ± 3.5 | 4.9 ± 2.3 | 0.741 |
| WBC (×10^9^/L) | 10.3 (7.2–13.8) | 11.8 (9.5–16.8) | 0.207 |
| **NLR** | 3.3 (2.1–5.2) | 11.1 (4.0–18.09) | **0.001** |
| **Platelets (×10^9^/L)** | 350.0 (264.8–467.5) | 254.0 (157.5–343.5) | **0.039** |
| **CRP (mg/L)** | 3.7 (1.8–8.7) | 25.5 (13.0–60.3) | **<0.001** |
| ALT (U/L) | 15.0(11.0–24.8) | 19.0 (10.5–78.0) | 0.339 |
| LDH (U/L) | 303.0 (260.8–360.8) | 606.0 (248.0–896.0) | 0.077 |
| PT (s) | 12.0 (11.5–13.1) | 12.9 (11.3–13.5) | 0.561 |
| **APTT (s)** | 31.6 (29.0–35.2) | 28.0 (26.0–30.9) | **0.023** |
| Fibrinogen (g/L) | 3.3 (2.8–3.7) | 2.6(2.4–3.4) | 0.055 |
| **D-dimer (ng/mL)** | 197.5 (135.5–341.0) | 4255.0 (756.0–9584.5) | **<0.001** |

Statistics: P values were calculated using the unpaired two-tailed Student’s *t* test, the Mann–Whitney *U* test, or Fisher’s exact test. Continuous variables are presented as mean ± standard deviation or median (interquartile range), and categorical variables are presented as number (percentage). PSM was performed using optimal matching with a caliper of 0.05.

Abbreviations: APTT, activated partial thromboplastin time; ALT, alanine aminotransferase; CI, cerebral infarction; CRP, C-reactive protein; LDH, lactate dehydrogenase; MPP, Mycoplasma pneumoniae pneumonia; NLR, neutrophil-to-lymphocyte ratio; WBC, white blood cell count.

Table 4. Multivariable Firth’s penalized logistic regression analysis in the propensity score–matched cohort using optimal matching

| Characteristics | OR | 95% CI | P |
| --- | --- | --- | --- |
| **CRP** | **1.114** | 1.016 – 1.328 | **0.008** |
| NLR | 1.122 | 0.896 – 1.423 | 0.302 |
| PLT | 1.001 | 0.990 – 1.012 | 0.801 |
| APTT | 0.655 | 0.285 – 1.073 | 0.103 |
| **D-dimer** | **1.00037** | 1.00014 – 1.00080 | **0.001** |

**Supplementary Table 5. Clinical Manifestations of the patient with CI**

| Case | 1 | 2 | 3 | 4 | 5 | 6 | 7 | 8 | 9 |
| --- | --- | --- | --- | --- | --- | --- | --- | --- | --- |
| Gender | Male | Male | Male | Male | Female | Male | Male | Male | Female |
| Age (years) | 5.1 | 12.8 | 5.9 | 6.3 | 5.2 | 12.8 | 9.8 | 5.8 | 3.7 |
| Respiratory Symptoms | Fever, cough | Fever, cough | Cough, fever | Cough, fever | Fever, cough | Fever | Fever, cough | Fever, cough | Fever, cough |
| Neurological Symptoms | Right limb weakness, dysarthria | Left limb weakness, vomiting, seizures | Lethargy, vomiting, seizures | Bilateral limb weakness, lethargy | Seizures | Right lower limb weakness | Left limb weakness, | seizures | unconsciousness |
| clinical course from onset to CI | 30d | 6d | 14d | 15d | 8d | 14d | 5d | 9d | 7d |
| CSF -MP-DNA | Negative | Negative | Negative | Negative | Negative | Negative | NA | Negative | NA |
| Chest X-ray/CT | Left-sided pneumonia | Right-sided pneumonia | Right-sided pneumonia | Bilateral pneumonia | Left-sided pneumonia | Left-sided pneumonia consolidation | Right-sided pneumonia | Right-sided pneumonia | Bilateral pneumonia |
| Cranial CT/MRI | Left frontal-temporal and basal ganglia | Right basal ganglia and temporal-occipital lobes, bilateral cerebellar hemispheres | Left temporal-occipital lobes | Bilateral basal ganglia, cerebral peduncles | Left frontal-temporal-parietal lobes and basal ganglia | Left parietal lobe | Right cerebral hemisphere | Multiple abnormal signals in the brain | Bilateral thalamus, brainstem, cerebellum |
| Magnetic resonance angiography. | NA | Bilateral anterior cerebral arteries, bilateral internal carotid arteries | NA | NA | Left anterior and middle cerebral arteries | Left superficial cerebral veins | Right middle cerebral artery | Basilar artery and bilateral posterior cerebral arteries | Posterior basilar artery and bilateral posterior cerebral arteries |
| Outcones | Motor disorders, facial paralysis | Reduced muscle strength in the left upper limb | Motor disorders | Motor disorders, epilepsy, mental retardation | Motor disorders, facial paralysis | Motor disorders | Death | Death | Death |

Abbreviations: CI, cerebral infarction; CSF, cerebrospinal fluid; MP, *Mycoplasma pneumoniae*; MRI, magnetic resonance imaging.
